# Association of 1,25 dihydroxyvitamin D with left ventricular hypertrophy and left ventricular diastolic dysfunction in patients with chronic kidney disease

**DOI:** 10.1371/journal.pone.0302849

**Published:** 2024-05-09

**Authors:** Jemin Hyeon, Suji Kim, Byung Min Ye, Seo Rin Kim, Dong Won Lee, Soo Bong Lee, Il Young Kim

**Affiliations:** 1 Department of Internal Medicine, Pusan National University School of Medicine, Yangsan, Republic of Korea; 2 Research Institute for Convergence of Biomedical Science and Technology, Pusan National University Yangsan Hospital, Yangsan, Republic of Korea; Istituto Di Ricerche Farmacologiche Mario Negri, ITALY

## Abstract

Left ventricular hypertrophy (LVH) and left ventricular diastolic dysfunction (LVDD) are highly prevalent predictors of cardiovascular disease in individuals with chronic kidney disease (CKD). Vitamin D, particularly 25-hydroxyvitamin D [25(OH)D], deficiency has been reported to be associated with cardiac structure and function in CKD patients. In the current study, we investigated the association between 1,25-dihydroxyvitamin D [1,25(OH)_2_D], the active form of 25(OH)D, and LVH/LVDD in CKD patients. We enrolled 513 non-dialysis CKD patients. The presence of LVH and LVDD was determined using transthoracic echocardiography. In multivariable analysis, serum 1,25(OH)_2_D levels, but not serum 25(OH)D, were independently associated with LVH [odds ratio (OR): 0.90, 95% confidential interval (CI): 0.88–0.93, P < 0.001]. Additionally, age, systolic blood pressure, and intact parathyroid hormone levels were independently associated with LVH. Similarly, multivariable analysis demonstrated that serum 1,25(OH)_2_D levels, but not 25(OH)D levels, were independently associated with LVDD (OR: 0.88, 95% CI: 0.86–0.91, P < 0.001) with systolic blood pressure showing independent association with LVDD. The optimal cut-off values for serum 1,25(OH)_2_D levels for identifying LVH and LVDD were determined as ≤ 12.7 pg/dl and ≤ 18.1 pg/dl, respectively. Our findings suggest that serum 1,25(OH)_2_D levels have independent association with LVH and LVDD in CKD patients, underscoring their potential as biomarkers for these conditions in this patient population.

## Introduction

Cardiovascular disease (CVD) stands as the primary cause of mortality among individuals grappling with chronic kidney disease (CKD) [1, 2]. Despite extensive research, the precise etiology of CVD in CKD patients remains elusive and is believed to stem from a complex interplay of various factors [3, 4]. Previous studies have demonstrated that not only traditional CVD risk factors but also CKD-related factors, including disorders in calcium-phosphate metabolism, secondary hyperparathyroidism, dysregulation of renin-angiotensin system (RAS), chronic inflammation, anemia, fibroblast growth factor 23 (FGF23), and homocysteinemia, are associated with CVD in these patients [3–5]. Additionally, structural and functional abnormalities of the heart are common in CKD patients and contribute significantly to the development of CVD [6]. Left ventricular hypertrophy (LVH) and left ventricular diastolic dysfunction (LVDD) are representative structural and functional abnormalities of the heart in CKD patients and are reported to be associated with increased cardiovascular mortality [4].

Vitamin D deficiency is common in CKD patients and is caused by a lack of 25-hydroxyvitamin D [25(OH)D] and impaired activation of 1α-hydroxylase, which converts 25(OH)D to 1,25 dihydroxyvitamin D [1,25(OH)_2_D] [7]. Previous studies have demonstrated that vitamin D deficiency is associated with increased mortality and adverse cardiovascular outcomes, not only in the general population but also in CKD patients [8]. Vitamin D deficiency is known to be associated with CVDs in CKD by modifying cardiac structure and function [9]. Previous studies have indicated an association between vitamin D deficiency and LVH/LVDD in CKD [10–12]. However, further research is needed to enhance our understanding of the connection between vitamin D deficiency and LVH/LVDD.

We have already published a study similar to the current one on LVH and LVDD in CKD patients, titled “Association between serum uric acid and left ventricular hypertrophy/left ventricular diastolic dysfunction in patients with chronic kidney disease” [13]. In the current study, we hypothesized that vitamin D deficiency, assessed by serum 25(OH)D and 1,25(OH)_2_D levels, is independently associated with LVH and LVDD in these patients. To test this hypothesis, we conducted a cross-sectional study to investigate the relationship between serum levels of 25(OH)D and 1,25(OH)_2_D, and LVH/LVDD in CKD patients.

## Materials and methods

### Study population

The current study involved a retrospective analysis of the medical records of 513 patients, aged 18 years and above, who had visited the Pusan National University Yangsan Hospital between 2010 and 2019, building upon the study cohort from a previous investigation [13]. We utilized the CKD Epidemiology Collaboration (CKD-EPI) equation [14] to determine the estimated glomerular filtration rate (eGFR). All participants included in the study were diagnosed with CKD (eGFR < 60 ml/min/1.73 m^2^) and were not undergoing dialysis. To ensure the exclusion of patients with acute kidney injury and to identify those with CKD, we restricted inclusion to patients with documented previous serum creatinine levels in their medical records or those who had been under observation for at least 3 months. Patients with valvular heart disease, congenital heart disease, cardiomyopathy, systolic heart failure (ejection fraction < 50%), and atrial fibrillation were excluded from the analysis. Additionally, individuals who had received vitamin D supplements, calcimimetics, or phosphate binders that could potentially influence endogenous vitamin D levels were also excluded. Approval for the study protocol was obtained from the Institutional Review Board of Pusan National University Yangsan Hospital (IRB No. 05-2022-223), and all research and data collection procedures adhered strictly to the Declaration of Helsinki and current ethical guidelines. Informed consent was waived by the Institutional Review Board due to the retrospective nature of the analysis, which relied solely on anonymized medical charts and records. Data retrieval occurred between October 20, 2022, and February 25, 2023.

### Study variables

Utilizing the study cohort established in a previous investigation [13], we collected demographic and clinical details including age, gender, diabetes status, and history of CVDs (such as coronary heart disease, cerebrovascular disease, and peripheral vascular disease). Additionally, information regarding concurrent medication usage, including angiotensin-converting enzyme inhibitors (ACEIs), angiotensin receptor blockers (ARBs), calcium channel blockers, beta-blockers, and thiazide/loop diuretics, was gathered. Body mass index was calculated using measurements of weight and height, expressed in kg/m^2^. Blood pressure readings were obtained from the upper right arm of each patient in a seated position using an automated sphygmomanometer following a 5-minute rest period [13]. Moreover, various blood parameters such as 25(OH)D, 1,25(OH)_2_D, albumin, calcium, phosphate, total cholesterol, hemoglobin, C-reactive protein (CRP), and intact parathyroid hormone (PTH) levels were concurrently assessed. Urinary albumin levels were determined by calculating the urinary albumin-to-creatinine ratio (mg/g Cr). Serum 25(OH)D levels were measured using chemiluminescence immunoassay (DiaSorin, Saluggia, Italy; reference range: 4.8–52.8 ng/ml; the inter-assay and intra-assay coefficients of variation were 1.0–1.4% and 2.4–5.7%). Serum 1,25(OH)_2_D levels were measured using radioimmunoassay (DIAsource ImmunoAssays, Louvain-la-Neuve, Belgium; reference range: 19.6–54.3 pg/ml; the inter-assay and intra-assay coefficients of variation were 3.1–5.9% and 3.6–6.6%). Throughout the study period from 2010 to 2019, our hospital’s laboratory maintained quality control certification from The Korean Association of External Quality Assessment Service.

### Echocardiography

In accordance with our previous study [13], transthoracic echocardiography was performed on all participants utilizing an IE33 echo system (Philips, Amsterdam, The Netherlands). Echocardiographic data were collected following the guidelines outlined by the American Society of Echocardiography [15] and were analyzed by a skilled cardiologist blinded to clinical information. Using the M-mode in the parasternal long-axis view, the left ventricular (LV) mass was estimated using the cube formula at end-diastole (LV mass = 0.8 × [1.04 × {interventricular septum thickness + LV internal diameter + posterior wall thickness}^3^ –{LV internal diameter}^3^] + 0.6 g) [13]. The LV mass index (LVMI) was calculated by dividing LV mass by body surface area (BSA) [LVMI = LV mass (g)/BSA (m^2^)] [13, 15]. LVH was defined as LVMI > 115 g/m^2^ in men and > 95 g/m^2^ in women [13, 15]. LV systolic function, represented by LV ejection fraction (LVEF), was calculated using the biplane Simpson’s method. LVDD assessment was conducted using Doppler echocardiography and tissue Doppler imaging. Early mitral inflow velocity (E) and late mitral inflow velocity (A) were measured via Doppler echocardiography [13, 15]. Peak early mitral annular velocity (e’) was determined as the average of velocities obtained at the medial and lateral annuli using tissue Doppler. The E/e’ ratio was then computed to estimate LV filling pressure [13]. LVDD severity was evaluated based on e’ value, E/e’ ratio, and left atrial volume index (LAVI). Following the guidelines of the American Society of Echocardiography [16], parameters such as e’, LAVI, E/A ratio, deceleration time of E, and E/e’ ratio were utilized to categorize LVDD into normal function or grades 1, 2, or 3. LVDD was defined as grade ≥ 1 dysfunction [13].

### Statistical analysis

Continuous variables were represented as mean values along with their standard deviations, while categorical variables were depicted as percentages. Group distinctions were assessed utilizing one-way analysis of variance for continuous variables and the chi-square test for categorical data. Pearson’s correlation coefficient was employed to explore the relationship between serum 1,25(OH)_2_D levels and echocardiographic findings. For the prediction of LVH and LVDD, univariable and multivariable logistic regression analyses were carried out to calculate the odds ratio (OR) along with its 95% confidence interval (CI) [17]. Significant variables were identified through univariable analysis, and for multivariable analysis, clinically pertinent variables were selected (including age, sex, diabetes, coronary artery disease, systolic blood pressure, diastolic blood pressure, eGFR, urinary albumin, phosphate, hemoglobin, CRP, intact PTH, and 1,25(OH)_2_D). The receiver operating characteristic (ROC) curve analysis was utilized to determine the area under the curve (AUC), with the Youden index utilized to identify the optimal cut-off value of serum 1,25(OH)_2_D levels for detecting LVH and LVDD among study participants. Statistical significance was set at P < 0.05. All statistical analyses were conducted using SPSS version 27.0 Statistical Package for the Social Sciences (SPSS, Inc., Chicago, IL, USA) and MedCalc Statistical Software version 19.7.2 (MedCalc Software, Ostend, Belgium).

## Results

### Baseline characteristics of the study subjects

Table 1 depicts the baseline variables of the study subjects stratified by CKD stage. Among the 513 patients, 272 were identified as having CKD stage 3, 181 as CKD stage 4, and 60 as CKD stage 5. Analysis of serum vitamin D levels revealed a trend of decreasing levels with advancing CKD stages, both for 25(OH)D and 1,25(OH)_2_D. Additionally, patients in higher CKD stages exhibited a higher LVMI and a greater prevalence of LVH. Moreover, individuals in higher CKD stages demonstrated an elevated E/e’ ratio, E/A ratio, and LAVI, along with lower values of e’ and A. Notably, no significant differences were observed among the three CKD groups regarding the prevalence of LVDD.

**Table 1:** Baseline characteristics of the study subjects according to CKD stage (n = 513).

	CKD stage 3	CKD stage 4	CKD stage 5	P
	(n = 272)	(n = 181)	(n = 60)	
Age (years)	57.2 ± 10.1	61.6 ± 10.0	62.1 ± 12.4	<0.001
Sex, male [n (%)]	152 (55.9%)	104 (57.5%)	35 (58.3%)	0.913
Diabetes [n (%)]	138 (50.7%)	95 (52.5%)	34 (56.7%)	0.700
Cardiovascular disease [n (%)]				
Coronary heart disease	42 (15.4%)	40 (22.1%)	17 (28.3%)	0.036
Cerebrovascular disease	19 (7.0%)	22 (12.2%)	11 (18.3%)	0.017
Peripheral vascular disease	14 (5.1%)	15 (8.3%)	9 (15.0%)	0.026
Medication [n (%)]				
ACEI or ARB	203 (74.6%)	141 (77.9%)	53 (88.3%)	0.070
Calcium channel blocker	155 (57.0%)	112 (61.9%)	41 (68.3%)	0.219
Beta blocker	82 (30.1%)	78 (43.1%)	38 (63.3%)	<0.001
Thiazide diuretics	100 (36.8%)	35 (19.3%)	8 (13.3%)	<0.001
Loop diuretics	95 (34.9%)	87 (48.1%)	34 (56.7%)	0.001
Body mass index (kg/m^2^)	23.7 ± 2.5	23.8 ± 2.7	23.2 ± 1.6	0.209
Systolic blood pressure (mmHg)	132.5 ± 17.5	134.7 ± 16.3	145.0 ± 18.9	<0.001
Diastolic blood pressure (mmHg)	78.8 ± 13.6	79.2 ± 13.6	82.1 ± 15.0	0.237
eGFR (ml/min/1.73 m^2^)	43.1 ± 8.5	22.2 ± 4.5	9.7 ± 2.4	<0.001
Urinary albumin (mg/g Cr)	1303.3 ± 850.3	2280.8 ± 936.2	3020.3 ± 1259.0	<0.001
Albumin (g/dl)	4.0 ± 0.3	3.8 ± 0.3	3.6 ± 0.4	<0.001
Calcium (mg/dl)	9.1 ± 0.4	9.0 ± 0.4	8.7 ± 0.4	<0.001
Phosphate (mg/dl)	3.4 ± 0.6	3.9 ± 0.8	5.0 ± 1.0	<0.001
Total cholesterol (mg/dl)	208.7 ± 41.1	206.3 ± 42.3	217.3 ± 43.6	0.892
Hemoglobin (g/dl)	12.7 ± 1.8	10.7 ± 1.8	9.5 ± 1.4	<0.001
C-reactive protein (mg/dl)	0.6 ± 0.5	0.7 ± 0.5	1.2 ± 1.2	<0.001
Intact parathyroid hormone (pg/ml)	55.8 ± 31.1	123.9 ± 70.1	209.2 ± 88.6	<0.001
25(OH)D (ng/ml)	24.1 ± 7.9	20.7 ± 7.6	19.3 ± 5.3	<0.001
1,25(OH)_2_D (pg/dl)	20.1 ± 10.6	16.7 ± 10.7	12.1 ± 7.9	<0.001
Left ventricular mass index (g/m^2^)	95.9 ± 23.8	106.9 ± 24.2	114.2 ± 22.2	<0.001
E (cm/s)	62.3 ± 8.3	61.6 ± 8.8	61.3 ± 9.8	0.575
A (cm/s)	64.2 ± 11.2	59.6 ± 9.4	57.3 ± 11.3	<0.001
E/A	0.99 ± 0.2	1.05 ± 0.2	1.13 ± 0.4	<0.001
e’ (cm/s)	8.0 ± 1.4	7.6 ± 1.2	7.3 ± 1.4	0.001
E/e’	8.1 ± 1.8	8.2 ± 1.4	8.8 ± 2.7	0.024
Left atrial volume index (ml/m^2^)	34.3 ± 6.8	35.5 ± 6.4	36.6 ± 7.8	0.027
Left ventricular ejection fraction (%)	62.3 ± 6.4	61.7 ± 7.6	62.1 ± 7.5	0.629
Left ventricular hypertrophy	81 (29.8%)	95 (52.5%)	39 (65.0%)	<0.001
Left ventricular diastolic dysfunction	162 (59.6%)	112 (61.9%)	39 (65.0%)	0.705

The data are expressed as either mean ± standard deviation or as proportions (n %). A; late mitral inflow velocity; E, early mitral inflow velocity; e, peak early mitral annular velocity.

**Fig 1:**
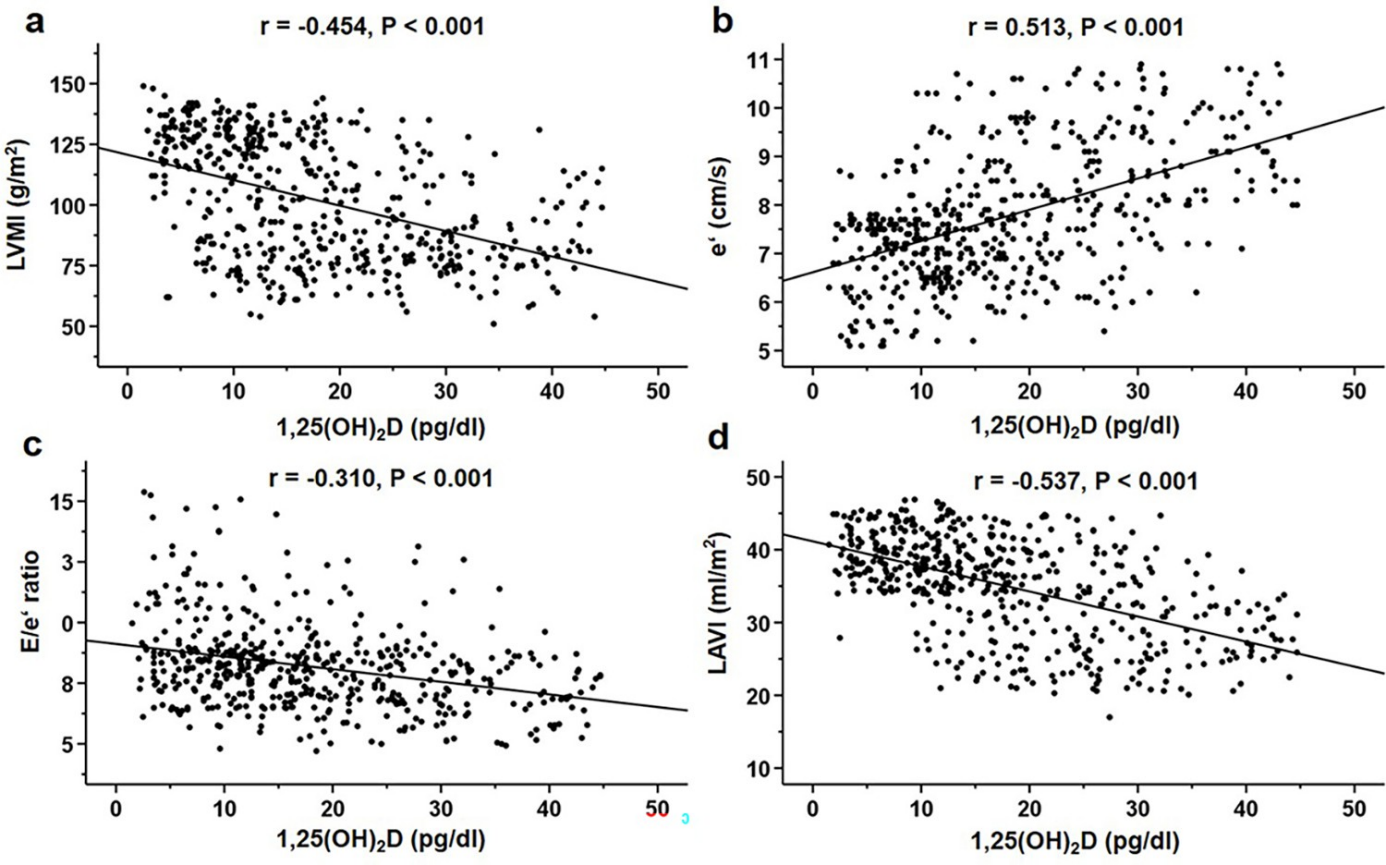
Correlations between serum 1,25(OH)_2_D levels and left ventricular mass index (a), e’ (b), E/e’ (d), and left atrial volume index (d) in the study subjects (n = 513). Serum 1,25(OH)_2_D levels correlated negatively with the left ventricular mass index, E/e’ ratio, and left atrial volume index and positively with e’. E, early mitral inflow velocity; e’, peak early mitral annular velocity; 1,25(OH)_2_D, 1,25-dihydroxyvitamin D.

Table 2 displays echocardiographic findings categorized by serum levels of 1,25(OH)_2_D. Patients in the highest tertile of serum 1,25(OH)_2_D were associated with significantly lower LVMI, E/e’ ratio, E/A ratio, and LAVI values, as well as higher e’ and A values. Additionally, they exhibited a reduced prevalence of LVH and LVDD. Fig 1 depicts the correlation between serum 1,25(OH)2D levels and LVMI, e’, E/e’ ratio, and LAVI. Serum 1,25(OH)_2_D levels displayed a negative correlation with LVMI, E/e’ ratio, and LAVI, while demonstrating a positive correlation with e’.

**Table 2:** Echocardiographic data according to tertiles of serum 1,25(OH)_2_D levels in the study subjects (n = 513).

	Tertile 1	Tertile 2	Tertile 3	P
	(<11.6 pg/dl)	(11.6–21.5 pg/dl)	(>21.6 pg/dl)	
Left ventricular mass index (g/m^2^)	115.8 ± 22.9	101.5 ± 24.3	88.1 ± 18.5	<0.001
E (cm/s)	61.2 ± 9.3	60.7 ± 7.9	63.9 ± 8.4	0.002
A (cm/s)	56.0 ± 8.3	60.6 ± 9.8	68.7 ± 10.7	<0.001
E/A	1.11 ± 0.3	1.02 ± 0.2	0.95 ± 0.2	<0.001
e’ (cm/s)	7.1 ± 1.0	7.6 ± 1.2	8.6 ± 1.3	<0.001
E/e’	8.8 ± 2.0	8.1 ± 1.6	7.6 ± 1.5	<0.001
Left atrial volume index (ml/m^2^)	39.1 ± 4.2	35.5 ± 6.6	30.3 ± 6.3	<0.001
Left ventricular ejection fraction (%)	62.0 ± 7.1	62.1 ± 6.8	62.2 ± 7.0	0.963
Left ventricular hypertrophy	123 (71.1%)	67 (39.0%)	25 (14.9%)	<0.001
Left ventricular diastolic dysfunction	148 (85.5%)	119 (69.2%)	46 (27.4%)	<0.001

The data are expressed as either mean ± standard deviation or as proportions (n %). A; late mitral inflow velocity; E, early mitral inflow velocity; e’, peak early mitral annular velocity.

### Association between serum 1,25(OH)_2_D levels and LVH

Table 3 presents the baseline factors correlated with LVH among the study participants. Upon univariable analysis, it was noted that serum levels of 1,25(OH)_2_D were independently associated with LVH, while no such association was found with serum 25(OH)D levels. Alongside serum 1,25(OH)_2_D levels, significant factors tied to LVH encompassed age, coronary heart disease, blood pressure, hemoglobin, urinary albumin levels, phosphate, eGFR, CRP, and intact PTH. Following multivariable analysis, it was evident that serum 1,25(OH)_2_D levels (OR: 0.90, 95% CI: 0.88–0.93; P < 0.001) sustained their independent association with LVH. Additionally, age, systolic blood pressure, and intact PTH emerged as independent factors correlated with LVH.

**Table 3:** Univariable and multivariable analyses for variables associated with left ventricular hypertrophy in the study subjects (n = 513).

	Univariable		Multivariable	
	OR (95% CI)	P	OR (95% CI)	P
Age (1 year)	1.05 (1.03–1.07)	<0.001	1.04 (1.02–1.07)	<0.001
Sex, male	0.91 (0.64–1.30)	0.908	0.75 (0.47–1.20)	0.226
Diabetes	0.86 (0.67–1.10)	0.231	0.95 (0.60–1.51)	0.829
Cardiovascular disease				
Coronary heart disease	1.62 (1.04–2.52)	0.032	1.52 (0.83–2.78)	0.171
Cerebrovascular disease	1.57 (0.88–2.79)	0.125		
Peripheral vascular disease	1.60 (0.82–3.10)	0.167		
Medication				
ACEI or ARB	0.94 (0.62–1.43)	0.767		
Calcium channel blocker	0.96 (0.67–1.38)	0.843		
Beta blocker	1.11 (0.77–1.59)	0.579		
Thiazide diuretics	0.82 (0.55–1.22)	0.820		
Loop diuretics	0.98 (0.69–1.40)	0.983		
Body mass index (1 kg/m^2^)	0.94 (0.88–1.01)	0.944		
Systolic blood pressure (10 mmHg)	1.76 (1.54–2.02)	<0.001	1.62 (1.38–1.92)	<0.001
Diastolic blood pressure (10 mmHg)	1.20 (1.05–1.36)	0.007	0.96 (0.80–1.03)	0.665
eGFR (1 ml/min/1.73 m^2^)	0.97 (0.95–0.98)	<0.001	1.00 (0.98–1.03)	0.762
Urinary albumin (100 mg/g Cr)	1.02 (1.00–1.03)	0.033	0.99 (0.97–1.02)	0.481
Albumin (1 g/dl)	0.84 (0.52–1.38)	0.844		
Calcium (1 mg/dl)	0.89 (0.59–1.36)	0.894		
Phosphate (1 mg/dl)	1.47 (1.20–1.81)	<0.001	0.98 (0.73–1.31)	0.890
Total cholesterol (1 mg/dl)	1.00 (1.00–1.00)	0.687		
Hemoglobin (1 g/dL)	0.86 (0.79–0.94)	0.001	1.02 (0.90–1.16)	0.792
C-reactive protein (1 mg/dL)	1.45 (1.09–1.93)	0.011	1.04 (0.73–1.49)	0.822
Intact parathyroid hormone (10 pg/ml)	1.11 (1.08–1.14)	<0.001	1.09 (1.04–1.14)	<0.001
25(OH)D (ng/ml)	1.00 (1.08–1.14)	0.775		
1,25(OH)_2_D (pg/dl)	0.89 (0.87–0.91)	<0.001	0.90 (0.88–0.93)	<0.001

Key variables were pinpointed through univariable analysis, and clinically relevant factors were chosen for multivariable analysis (age, sex, diabetes, coronary artery disease, systolic blood pressure, diastolic blood pressure, eGFR, urinary albumin, phosphate, hemoglobin, CRP, intact PTH, and 1,25(OH)_2_D).

### Association between serum 1,25(OH)_2_D levels and LVDD

Table 4 displays the variables linked to LVDD in the study cohort Univariable analysis revealed that serum levels of 1,25(OH)2D, in contrast to 25(OH)D levels, displayed an independent correlation with LVDD. Moreover, age, systolic blood pressure, eGFR, and intact PTH demonstrated significant associations with LVDD. Upon conducting multivariable analysis, serum 1,25(OH)_2_D levels (OR: 0.88, 95% CI: 0.86–0.91, P < 0.001) were independently associated with LVDD. Additionally, systolic blood pressure maintained its independent association with the presence of LVDD.

**Table 4:** Univariable and multivariable analyses for variables associated with left ventricular diastolic dysfunction in the study subjects (n = 513).

	Univariable		Multivariable	
	OR (95% CI)	P	OR (95% CI)	P
Age (1 year)	1.03 (1.01–1.05)	0.002	1.02 (1.00–1.04)	0.125
Sex, male	0.98 (0.69–1.41)	0.982	0.92 (0.59–1.44)	0.922
Diabetes	1.18 (0.83–1.69)	0.356	1.44 (0.92–2.25)	0.108
Cardiovascular disease				
Coronary heart disease	0.76 (0.49–1.20)	0.216		
Cerebrovascular disease	0.66 (0.37–1.20)	0.158		
Peripheral vascular disease	1.42 (0.70–2.88)	0.333		
Medication				
ACEI or ARB	1.09 (0.71–1.66)	0.701		
Calcium channel blocker	0.82 (0.57–1.17)	0.274		
Beta blocker	0.94 (0.65–1.35)	0.737		
Thiazide diuretics	0.84 (0.57–1.25)	0.391		
Loop diuretics	1.04 (0.73–1.50)	0.824		
Body mass index (1 kg/m^2^)	0.99 (0.92–1.06)	0.724		
Systolic blood pressure (10 mmHg)	1.35 (1.21–1.51)	<0.001	1.23 (1.07–1.42)	0.005
Diastolic blood pressure (10 mmHg)	1.14 (1.00–1.29)	0.054	1.01 (0.84–1.20)	0.951
eGFR (1 ml/min/1.73 m^2^)	0.99 (0.98–1.00)	0.032	1.01 (0.99–1.03)	0.384
Urinary albumin (100 mg/g Cr)	1.01 (0.99–1.03)	0.252		
Albumin (1 g/dl)	0.79 (0.48–1.32)	0.371		
Calcium (1 mg/dl)	0.94 (0.61–1.44)	0.776		
Phosphate (1 mg/dl)	1.13 (0.92–1.40)	0.236		
Total cholesterol (1 mg/dl)	1.00 (1.00–1.01)	0.729		
Hemoglobin (1 g/dl)	0.95 (0.88–1.04)	0.258		
C-reactive protein (1 mg/dl)	1.25 (0.93–1.68)	0.135		
Intact parathyroid hormone (10 pg/ml)	1.04 (1.02–1.07)	0.001	1.01 (0.98–1.05)	0.506
25(OH)D (ng/ml)	1.01 (0.98–1.03)	0.687		
1,25(OH)_2_D (pg/dl)	1.01 (0.86–0.90)	<0.001	0.88 (0.86–0.91)	<0.001

Key variables were pinpointed through univariable analysis, and clinically relevant factors were chosen for multivariable analysis (age, sex, diabetes, systolic blood pressure, diastolic blood pressure, eGFR, intact PTH, and 1,25(OH)_2_D).

### Performance of serum 1,25(OH)_2_D levels for detecting LVH and LVDD

**Fig 2:**
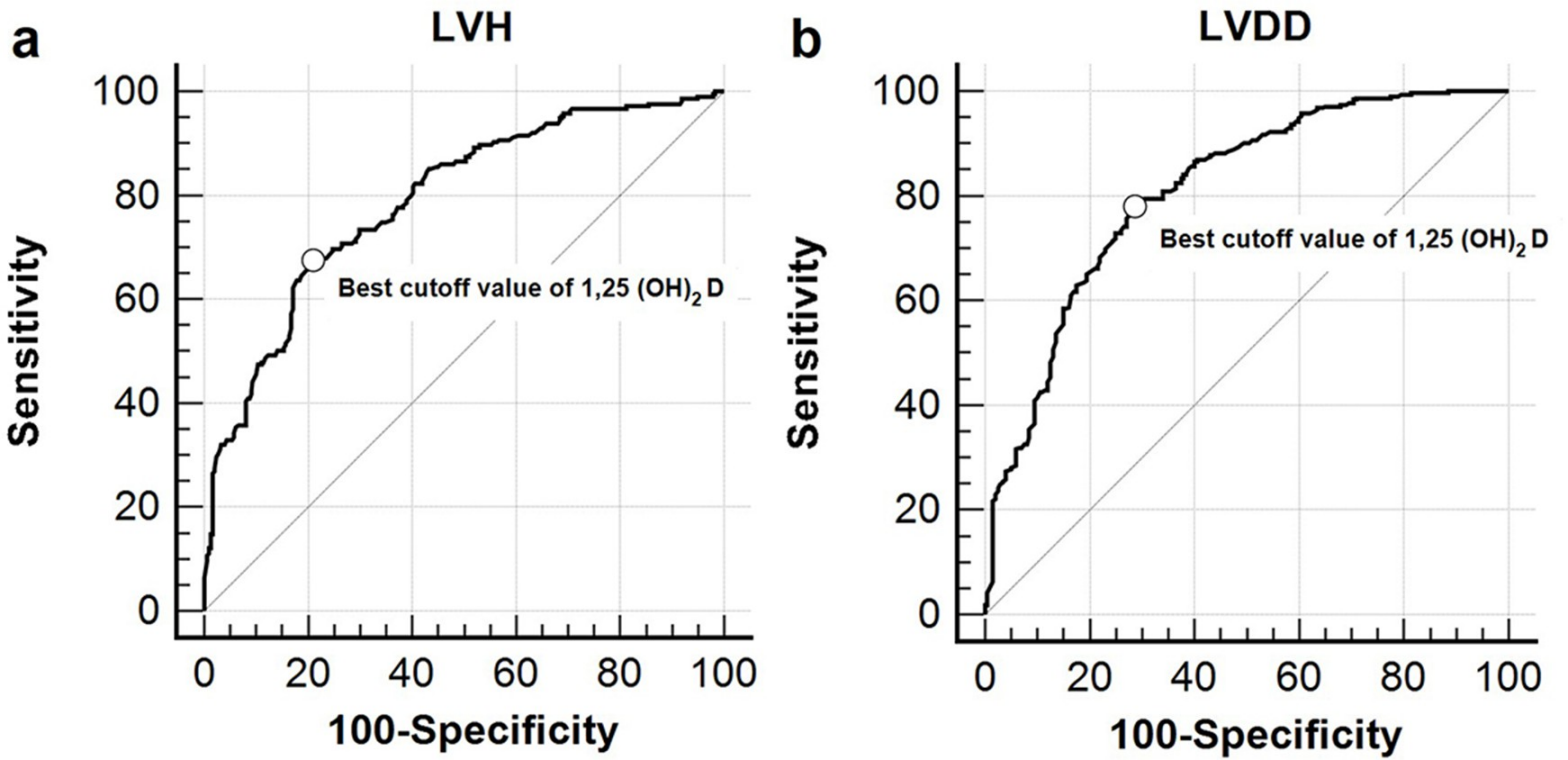
ROC curves depicting serum 1,25(OH)_2_D levels’ predictive ability for detecting left ventricular hypertrophy (a) and left ventricular diastolic dysfunction (b) in the study subjects (n = 513). (a) The AUC for serum 1,25(OH)_2_D levels in predicting left ventricular hypertrophy was 0.791 (95% CI: 0.753–0.825, p < 0.001). The optimal cut-off value for serum 1,25(OH)_2_D levels to predict the presence of left ventricular hypertrophy was ≤ 12.7 pg/dl, yielding a sensitivity of 67.4% (95% CI: 60.7–73.7%) and specificity of 79.2% (95% CI: 74.0–83.7%). (b) The AUC for serum 1,25(OH)_2_D levels in predicting left ventricular diastolic dysfunction was 0.810 (95% CI: 0.773–0.843, p < 0.001). The optimal cut-off value for serum 1,25(OH)_2_D levels to predict the presence of left ventricular diastolic dysfunction was ≤ 18.1 pg/dl, with a sensitivity of 78.0% (95% CI: 72.9–82.4%) and specificity of 71.5% (95% CI: 64.7–77.6%). AUC, area under the curve; CI, confidence interval; ROC, receiver-operating characteristic curves; 1,25(OH)_2_D, 1,25-dihydroxyvitamin D.

ROC analysis was carried out to evaluate the predictive ability of serum 1,25(OH)2D levels for detecting LVH and LVDD among study participants (Fig 2). Regarding LVH, the AUC for serum 1,25(OH)_2_D levels stood at 0.791 (95% CI: 0.753–0.825, p < 0.001). The optimal threshold value for serum 1,25(OH)_2_D levels to predict LVH presence was found to be ≤ 12.7 pg/dl, with a corresponding sensitivity of 67.4% (95% CI: 60.7–73.7%) and specificity of 79.2% (95% CI: 74.0–83.7%). As for LVDD, the AUC for serum 1,25(OH)_2_D level was determined as 0.810 (95% CI: 0.773–0.843, p < 0.001). The optimal threshold value for serum 1,25(OH)_2_D levels to predict LVDD presence was ≤ 18.1 pg/dl, with an associated sensitivity of 78.0% (95% CI: 72.9–82.4%) and specificity of 71.5% (95% CI: 64.7–77.6%).

## Discussion

In our present investigation, we explored the prevalence of LVH/LVDD across diverse stages of CKD. LVH prevalence demonstrated an escalating pattern in tandem with CKD stage progression. While LVDD prevalence tended to rise with advancing CKD stages, the disparity lacked statistical significance among the three CKD stages. Furthermore, the severity of both LVH and LVDD amplified as CKD stages advanced. Patients in more advanced CKD stages exhibited heightened LVMI and E/e’ ratio alongside a reduced e’ value. Notably, our study underscored the independent correlation of serum 1,25(OH)_2_D levels, rather than 25(OH)D, with LVH and LVDD among CKD patients. We also established the optimal cut-off value for serum 1,25(OH)_2_D levels in detecting LVH and LVDD.

The primary discovery of this research lies in the independent correlation observed between elevated serum 1,25(OH)_2_D levels and LVH and LVDD among CKD patients. While previous studies have also explored the link between vitamin D levels and LVH/LVDD in CKD patients, they were constrained by small sample sizes and inconclusive outcomes [18–20]. For instance, Patange et al. observed an association between 25(OH)D deficiency and LVH and LVDD in a cohort of 34 pediatric CKD patients [19]. Conversely, García-Cantón et al. found no significant association between 25(OH)D deficiency and LVH in a study involving 171 adult CKD patients [18]. Similarly, Sonkar et al. demonstrated no correlation between serum 25(OH)D levels and LVH or LVDD in an analysis of 86 adult CKD patients [20]. Corresponding with the conclusions of the aforementioned studies conducted on adult CKD patients [18, 20], our present investigation also did not reveal an association between serum 25(OH)D levels and LVH/LVDD in the multivariable analysis. However, our analysis did establish an independent association between serum 1,25(OH)_2_D levels and LVH/LVDD among CKD patients. Regarding the relationship between 1,25(OH)_2_D levels and LVH/LVDD, prior studies have explored these connections within the CKD population. In the Chronic Renal Insufficiency Cohort study, which investigated the associations of 25(OH)D and 1,25(OH)_2_D levels with CVD in CKD patients, serum 25(OH)D levels were associated with LVMI [10]. According to Ky et al.’s report, both 25(OH)D and 1,25(OH)_2_D deficiencies are associated with increased LV mass and LV volume [11]. In children with CKD, 25(OH)D levels, not 1,25(OH)_2_D levels, were independently associated with LVH [12]. Thus, our study contributes to the growing body of research supporting the association of vitamin D deficiency with LVH/LVDD in CKD patients.

LVH and LVDD exhibit a close interconnection, with LVH accompanied by myocardial fibrosis being the primary instigator of LVDD, resulting in heightened myocardial rigidity and compromised cardiac function during diastole [13, 21]. Among individuals with CKD, LVH and LVDD represent physiological responses to continual pressure and volume overload [13, 21]. Notably, factors associated with uremia, such as anemia and secondary hyperparathyroidism, alongside hypertension stemming from persistent pressure/volume overload, are posited to contribute to the underlying mechanisms driving LVH and LVDD development in CKD patients [13, 21]. In line with these suggested mechanisms, our current investigation underscores the independent associations of systolic blood pressure and intact PTH levels with LVH and LVDD in CKD patients. In our study cohort, while the prevalence of LVH and LVDD stood at 29.8% and 59.6% in CKD stage 3, respectively, these rates surged to 65% for both LVH and LVDD in CKD stage 5. Despite LVH typically being reported to precede LVDD, our findings suggest that LVDD might manifest before LVH in the earlier stages of CKD. The reason for this remains unclear, but factors related to CKD, aside from LVH, may influence the occurrence of LVDD. Previous studies have proposed that LVDD could be influenced by an increase in LV preload due to progression of renal dysfunction in CKD [21, 22]. Additionally, neurohumoral alterations, inflammation, anemia, and mineral disorders are thought to contribute to the development of LVDD [21, 22].

It is unclear how serum 1,25(OH)_2_D levels are associated with LVH and LVDD in CKD patients. Previous studies have suggested potential mechanisms for this association. First, hypertension and secondary hyperparathyroidism may affect the relationship between 1,25(OH)_2_D and LVH/LVDD [23, 24]. Vitamin D deficiency is reportedly associated with hypertension via modulation of the RAS [23]. Vitamin D deficiency also triggers secondary hyperparathyroidism in CKD, may directly promote LVH and LVDD development [23]. As mentioned above, the present study demonstrated that systolic blood pressure and intact PTH are independently associated with LVH and LVDD in CKD patients. However, the results of our multivariable analysis revealed that the association between serum 1,25(OH)_2_D levels and LVH/LVDD was independent of blood pressure and intact PTH levels, suggesting that this association might be independent of hypertension and secondary hyperparathyroidism. Second, vitamin D deficiency influences the progression of LVH and LVDD in CKD. The vitamin D receptor (VDR) has a wide tissue distribution, including cardiac tissues [3]. Vitamin D has been suggested to have several biological effects on cardiac tissues, including cardiac cell contraction, proliferation, maturation, and protein expression [3]. Cardiac hypertrophy has been observed in the hearts of VDR-knockout mice, suggesting that vitamin D may affect the myocardium directly and play a role in the development of myocyte hypertrophy [24, 25]. Activated vitamin D attenuated LV abnormalities induced by dietary sodium in salt-sensitive hypertensive rats [24, 26]. Furthermore, the selective VDR agonist paricalcitol suppressed the genetic expression of RAS in the experimental CKD model, and early administration of paricalcitol successfully halted histologic and functional deterioration of the kidney [27]. Altogether, these experimental findings suggest that vitamin D deficiency could directly contribute to cardiac structure and function abnormalities, including LVH and LVDD, thereby, increasing cardiovascular risk.

However, despite epidemiological and experimental evidence supporting the association between vitamin D deficiency and LVH/LVDD, including the results of the present study, vitamin D therapy does not seem to alter the progression of LVMI or LVDD in CKD patients. In the PRIMO randomized controlled trial, 48 weeks of therapy with active vitamin D and paricalcitol did not alter LVMI or LVDD in CKD patients [28]. In the OPERA trial, 52 weeks of therapy with paricalcitol improved secondary hyperparathyroidism; however, it did not alter the measures of LV structure and function in CKD patients [29]. In contrast, some studies have suggested that VDR activator therapy is associated with improved cardiac structure or function in CKD. A post hoc analysis of the PRIMO trial indicated that paricalcitol therapy was linked to a significant decrease in left atrial volume in patients with LVH and CKD [30]. Additionally, paricalcitol therapy was reported to correlate inversely with cardiac mass in uremic rats and hemodialysis patients [31]. Furthermore, intravenous calcitriol therapy was found to induce regression of myocardial hypertrophy in hemodialysis patients [32]. In conclusion, additional randomized controlled studies are warranted to confirm the beneficial effects of vitamin D therapy on LVH and LVDD in CKD patients.

There are several limitations inherent in this study. First, due to its retrospective and cross-sectional nature, establishing a temporal relationship and causality between serum 1,25(OH)_2_D and LVH/LVDD is challenging. Future investigations are warranted to delineate a causal link between 1,25(OH)_2_D and LVH/LVDD in CKD patients. Second, the stringent selection criteria employed in this study limit its generalizability. We specifically recruited individuals with maintained LV systolic function, excluding those with pre-existing heart conditions, to clarify the association between 1,25(OH)2D and LVH/LVDD. Consequently, the applicability of our findings to all CKD patients remains uncertain. Third, being a cross-sectional analysis exploring the correlation between vitamin D and echocardiography outcomes, the timing of vitamin D level assessment and echocardiography is crucial. However, not all serum 1,25(OH)_2_D levels were measured concurrently with echocardiographic examinations. The mean time between the measurement of serum 1,25(OH)_2_D levels and the echocardiographic assessment was 6.6 ± 4.1 days (range: 0–13 days). Finally, the absence of FGF23 measurements is a notable limitation of the current study. FGF23, which inhibits 1,25(OH)_2_D synthesis, is reported as a major contributor to myocardial hypertrophy in CKD [11]. It is noteworthy that a significant interaction by FGF23 on the relationship between vitamin D and LVH was reported, with patients exhibiting elevated FGF23 levels experiencing greater effects on LVH and adverse cardiac remodeling [11]. Similarly, vitamin D deficiency amplified the risk of adverse cardiac remodeling observed with elevated FGF23 [11]. Therefore, we believe that FGF23 must have influenced the association between vitamin D deficiency and LVH/LVDD in the current study. Consequently, we suggest that including FGF23 in the current study would provide clearer insights into the association between vitamin D deficiency and LVH/LVDD in CKD patients.

Despite these limitations, our study has several strengths. First, we investigated serum 1,25(OH)_2_D and 25(OH)D levels and found that serum 1,25(OH)_2_D levels, rather than 25(OH)D, were independently correlated with LVH and LVDD in CKD patients. This is significant because 1,25(OH)_2_D binds to the VDR and activates it, while 25(OH)D reflects vitamin D stores, being the main circulating form of vitamin D [33]. Thus, we believe that the results of the present study are more physiologically relevant than those of previous studies that assessed only 25(OH)D levels. Second, to uncover the connection between vitamin D and LVH/LVDD, the present study included only patients who were naïve to vitamin D supplements, calcimimetics, or phosphate binders that could affect endogenous vitamin D levels. Nevertheless, we cannot dismiss the possibility that some patients may have consumed natural preparations containing vitamin D. Third, this study’s findings carry significant clinical implications concerning cardiorenal syndrome (CRS), a term describing situations where dysfunction in either the heart or the kidney triggers dysfunction in the other [34]. Specifically, Type IV CRS, delineated as chronic reno-cardiac disease, manifests as primary CKD leading to cardiac dysfunction through mechanisms such as cardiac remodeling, LVH, LVDD, or heightened cardiovascular risk [34]. Our study not only established an independent correlation between serum 1,25(OH)_2_D levels and LVH/LVDD but also determined the optimal threshold of serum 1,25(OH)_2_D levels for detecting LVH and LVDD in CKD patients. Hence, we propose that serum 1,25(OH)_2_D levels could serve as a biomarker for LVH and LVDD in Type IV CRS.

## Conclusions

The current research established an independent correlation between serum levels of 1,25(OH)_2_D and both LVH and LVDD among individuals with CKD. Additionally, we identified the optimal threshold for serum 1,25(OH)_2_D levels in predicting the occurrence of LVH and LVDD, indicating its potential as a biomarker for these conditions in CKD patients. Further investigations are warranted to elucidate the involvement of vitamin D in the progression of LVH and LVDD among CKD patients.

## References

[1] Go AS, Chertow GM, Fan D, McCulloch CE, Hsu CY. Chronic kidney disease and the risks of death, cardiovascular events, and hospitalization. N Engl J Med. 2004; 351: 1296–305. doi: 10.1056/NEJMoa041031 15385656

[2] Jung HH. Albuminuria, estimated glomerular filtration rate, and traditional predictors for composite cardiovascular and kidney outcome: a population-based cohort study in Korea. Kidney Res Clin Pract. 2022; 41: 567–79. doi: 10.23876/j.krcp.22.005 35545220 PMC9576456

[3] Achinger SG, Ayus JC. The role of vitamin D in left ventricular hypertrophy and cardiac function. Kidney Int Suppl. 2005; S37–42. doi: 10.1111/j.1523-1755.2005.09506.x 15882312

[4] Farshid A, Pathak R, Shadbolt B, Arnolda L, Talaulikar G. Diastolic function is a strong predictor of mortality in patients with chronic kidney disease. BMC Nephrol. 2013; 14: 280. doi: 10.1186/1471-2369-14-280 24359445 PMC3878021

[5] Gutierrez OM, Mannstadt M, Isakova T, Rauh-Hain JA, Tamez H, Shah A, et al. Fibroblast growth factor 23 and mortality among patients undergoing hemodialysis. N Engl J Med. 2008; 359: 584–92. doi: 10.1056/NEJMoa0706130 18687639 PMC2890264

[6] Ronco C, McCullough P, Anker SD, Anand I, Aspromonte N, Bagshaw SM, et al. Cardio-renal syndromes: report from the consensus conference of the acute dialysis quality initiative. Eur Heart J. 2010; 31: 703–11. doi: 10.1093/eurheartj/ehp507 20037146 PMC2838681

[7] Kalkwarf HJ, Denburg MR, Strife CF, Zemel BS, Foerster DL, Wetzsteon RJ, et al. Vitamin D deficiency is common in children and adolescents with chronic kidney disease. Kidney Int. 2012; 81: 690–7. doi: 10.1038/ki.2011.431 22205356 PMC3634332

[8] Drechsler C, Pilz S, Obermayer-Pietsch B, Verduijn M, Tomaschitz A, Krane V, et al. Vitamin D deficiency is associated with sudden cardiac death, combined cardiovascular events, and mortality in haemodialysis patients. Eur Heart J. 2010; 31: 2253–61. doi: 10.1093/eurheartj/ehq246 20688781 PMC2938469

[9] Lai S, Coppola B, Dimko M, Galani A, Innico G, Frassetti N, et al. Vitamin D deficiency, insulin resistance, and ventricular hypertrophy in the early stages of chronic kidney disease. Ren Fail. 2014; 36: 58–64. doi: 10.3109/0886022X.2013.832308 24028070

[10] Hsu S, Zelnick LR, Bansal N, Brown J, Denburg M, Feldman HI, et al. Vitamin D Metabolites and Risk of Cardiovascular Disease in Chronic Kidney Disease: The CRIC Study. J Am Heart Assoc. 2023; 12: e028561. 37421259 10.1161/JAHA.122.028561 PMC10382125

[11] Ky B, Shults J, Keane MG, Sutton MS, Wolf M, Feldman HI, et al. FGF23 modifies the relationship between vitamin D and cardiac remodeling. Circ Heart Fail. 2013; 6: 817–24. doi: 10.1161/CIRCHEARTFAILURE.112.000105 23748358 PMC3867268

[12] Mitsnefes MM, Betoko A, Schneider MF, Salusky IB, Wolf MS, Juppner H, et al. FGF23 and Left Ventricular Hypertrophy in Children with CKD. Clin J Am Soc Nephrol. 2018; 13: 45–52. doi: 10.2215/CJN.02110217 29025789 PMC5753303

[13] Kim IY, Ye BM, Kim MJ, Kim SR, Lee DW, Kim HJ, et al. Association between serum uric acid and left ventricular hypertrophy/left ventricular diastolic dysfunction in patients with chronic kidney disease. PLoS One. 2021; 16: e0251333. doi: 10.1371/journal.pone.0251333 33956863 PMC8101764

[14] Levey AS, Stevens LA, Schmid CH, Zhang YL, Castro AF 3rdo, Feldman HI, et al. A new equation to estimate glomerular filtration rate. Ann Intern Med. 2009; 150: 604–12. doi: 10.7326/0003-4819-150-9-200905050-00006 19414839 PMC2763564

[15] Lang RM, Badano LP, Mor-Avi V, Afilalo J, Armstrong A, Ernande L, et al. Recommendations for cardiac chamber quantification by echocardiography in adults: an update from the American Society of Echocardiography and the European Association of Cardiovascular Imaging. J Am Soc Echocardiogr. 2015; 28: 1–39 e14. doi: 10.1016/j.echo.2014.10.003 25559473

[16] Nagueh SF, Appleton CP, Gillebert TC, Marino PN, Oh JK, Smiseth OA, et al. Recommendations for the evaluation of left ventricular diastolic function by echocardiography. J Am Soc Echocardiogr. 2009; 22: 107–33. doi: 10.1016/j.echo.2008.11.023 19187853

[17] Kim IY, Kim JH, Kim MJ, Lee DW, Hwang CG, Han M, et al. Plasma neutrophil gelatinase-associated lipocalin is independently associated with left ventricular hypertrophy and diastolic dysfunction in patients with chronic kidney disease. PLoS One. 2018; 13: e0205848. doi: 10.1371/journal.pone.0205848 30325973 PMC6191140

[18] Garcia-Canton C, Bosch E, Auyanet I, Ramirez A, Rossique P, Culebras C, et al. [25 hydroxyvitamin D levels and cardiovascular risk in a cohort of patients with advanced chronic kidney disease]. Nefrologia. 2010; 30: 435–42. 20651885 10.3265/Nefrologia.pre2010.Mar.10288

[19] Patange AR, Valentini RP, Gothe MP, Du W, Pettersen MD. Vitamin D deficiency is associated with increased left ventricular mass and diastolic dysfunction in children with chronic kidney disease. Pediatr Cardiol. 2013; 34: 536–42. doi: 10.1007/s00246-012-0489-z 22941497

[20] Sonkar SK, Bhutani M, Sonkar GK, Pandey SK, Chandra S, Bhosale V. Vitamin D levels and other biochemical parameters of mineral bone disorders and their association with diastolic dysfunction and left ventricular mass in young nondiabetic adult patients with chronic kidney disease. Saudi J Kidney Dis Transpl. 2017; 28: 758–63. 28748877

[21] Ogawa T, Nitta K. Clinical Impact of Left Ventricular Diastolic Dysfunction in Chronic Kidney Disease. Contrib Nephrol. 2018; 195: 81–91. doi: 10.1159/000486938 29734153

[22] de Simone G, Mancusi C. Diastolic function in chronic kidney disease. Clin Kidney J. 2023; 16: 1925–35. doi: 10.1093/ckj/sfad177 37915916 PMC10616497

[23] Covic A, Voroneanu L, Goldsmith D. The effects of vitamin D therapy on left ventricular structure and function—are these the underlying explanations for improved CKD patient survival? Nephron Clin Pract. 2010; 116: c187–95. doi: 10.1159/000317198 20606478

[24] Artaza JN, Mehrotra R, Norris KC. Vitamin D and the cardiovascular system. Clin J Am Soc Nephrol. 2009; 4: 1515–22. doi: 10.2215/CJN.02260409 19696220

[25] Xiang W, Kong J, Chen S, Cao LP, Qiao G, Zheng W, et al. Cardiac hypertrophy in vitamin D receptor knockout mice: role of the systemic and cardiac renin-angiotensin systems. Am J Physiol Endocrinol Metab. 2005; 288: E125–32. doi: 10.1152/ajpendo.00224.2004 15367398

[26] Bodyak N, Ayus JC, Achinger S, Shivalingappa V, Ke Q, Chen YS, et al. Activated vitamin D attenuates left ventricular abnormalities induced by dietary sodium in Dahl salt-sensitive animals. Proc Natl Acad Sci U S A. 2007; 104: 16810–5. doi: 10.1073/pnas.0611202104 17942703 PMC2040477

[27] Freundlich M, Quiroz Y, Zhang Z, Zhang Y, Bravo Y, Weisinger JR, et al. Suppression of renin-angiotensin gene expression in the kidney by paricalcitol. Kidney Int. 2008; 74: 1394–402. doi: 10.1038/ki.2008.408 18813285

[28] Thadhani R, Appelbaum E, Pritchett Y, Chang Y, Wenger J, Tamez H, et al. Vitamin D therapy and cardiac structure and function in patients with chronic kidney disease: the PRIMO randomized controlled trial. JAMA. 2012; 307: 674–84. doi: 10.1001/jama.2012.120 22337679

[29] Wang AY, Fang F, Chan J, Wen YY, Qing S, Chan IH, et al. Effect of paricalcitol on left ventricular mass and function in CKD—the OPERA trial. J Am Soc Nephrol. 2014; 25: 175–86. doi: 10.1681/ASN.2013010103 24052631 PMC3871774

[30] Tamez H, Zoccali C, Packham D, Wenger J, Bhan I, Appelbaum E, et al. Vitamin D reduces left atrial volume in patients with left ventricular hypertrophy and chronic kidney disease. Am Heart J. 2012; 164: 902–9 e2. doi: 10.1016/j.ahj.2012.09.018 23194491

[31] Czaya B, Seeherunvong W, Singh S, Yanucil C, Ruiz P, Quiroz Y, et al. Cardioprotective Effects of Paricalcitol Alone and in Combination With FGF23 Receptor Inhibition in Chronic Renal Failure: Experimental and Clinical Studies. Am J Hypertens. 2019; 32: 34–44. doi: 10.1093/ajh/hpy154 30329020 PMC6284753

[32] Kim HW, Park CW, Shin YS, Kim YS, Shin SJ, Kim YS, et al. Calcitriol regresses cardiac hypertrophy and QT dispersion in secondary hyperparathyroidism on hemodialysis. Nephron Clin Pract. 2006; 102: c21–9. doi: 10.1159/000088295 16166802

[33] Kim IY, Ye BM, Kim MJ, Kim SR, Lee DW, Kim HJ, et al. 1,25-dihydroxyvitamin D deficiency is independently associated with cardiac valve calcification in patients with chronic kidney disease. Sci Rep. 2022; 12: 915. doi: 10.1038/s41598-022-04981-x 35042976 PMC8766529

[34] House AA, Anand I, Bellomo R, Cruz D, Bobek I, Anker SD, et a l. Definition and classification of Cardio-Renal Syndromes: workgroup statements from the 7th ADQI Consensus Conference. Nephrol Dial Transplant. 2010; 25: 1416–20. 10.1093/ndt/gfq136 20228069

